# The Role of Parietal Epithelial Cells in the Pathogenesis of Podocytopathy

**DOI:** 10.3389/fphys.2022.832772

**Published:** 2022-03-11

**Authors:** Zhi-hang Li, Xiao-yan Guo, Xiao-ying Quan, Chen Yang, Ze-jian Liu, Hong-yong Su, Ning An, Hua-feng Liu

**Affiliations:** Key Laboratory of Prevention and Management of Chronic Kidney Disease of Zhanjiang City, Institute of Nephrology, Affiliated Hospital of Guangdong Medical University, Zhanjiang, China

**Keywords:** parietal epithelial cells, podocyte, signaling pathway, podocytopathy, glomerular

## Abstract

Podocytopathy is the most common feature of glomerular disorder characterized by podocyte injury- or dysfunction-induced excessive proteinuria, which ultimately develops into glomerulosclerosis and results in persistent loss of renal function. Due to the lack of self-renewal ability of podocytes, mild podocyte depletion triggers replacement and repair processes mostly driven by stem cells or resident parietal epithelial cells (PECs). In contrast, when podocyte recovery fails, activated PECs contribute to the establishment of glomerular lesions. Increasing evidence suggests that PECs, more than just bystanders, have a crucial role in various podocytopathies, including minimal change disease, focal segmental glomerulosclerosis, membranous nephropathy, diabetic nephropathy, IgA nephropathy, and lupus podocytopathy. In this review, we attempt to dissect the diverse role of PECs in the pathogenesis of podocytopathy based on currently available information.

## Introduction

Glomerular visceral epithelial cells also referred to as podocytes, are terminally differentiated cells found in the human body, containing a nucleus, and involved in primary processes, as well as foot processes (FPs). Podocytes, together with glomerular basement membrane (GBM) and glomerular endothelial cells, constitute a glomerular filtration barrier (GFB) to selectively filter primary urine from plasma. The selective permeability of GFB is dependent on sophisticatedly established slit diaphragms (SDs) formed by FPs of neighboring podocytes. Mature podocytes express some specific proteins necessary for their functions, such as podocalyxin and glomerular epithelial protein-1 (GLEPP-1) on the plasma membrane, nephrin, podocin and CD2-associated protein (CD2AP) associated with SDs, and synaptopodin and α-actinin-4 associated with the cytoskeleton (Bariety et al., 2006). The transcription factor wilms tumor protein 1 (WT-1) is also specifically and highly expressed in mature podocytes. Abnormalities in genetic factors, immune response, and metabolic stimulation disrupt the expression of these functional proteins, resulting in podocyte dysfunction and loss, which is the underlying reason for podocytopathies.

Bowman’s capsule, constituted by the expansion and depression of the proximal end of the renal tubules, is a double globular cyst with the inner layer as the visceral layer (podocyte), and the wall layer is comprised of Bowman’s basement membrane (BBM) and PECs. The body size of PEC varies from 0.1–0.3 microns in thickness and increases to 2.0–3.5 microns at the nucleus, with microvilli and cilia lining its surface (Ohse et al., 2009b). Predominantly due to a lack of specific marker proteins, PECs, one of the intrinsic cells in the glomerulus, have not been recognized. So far, several marker proteins have been confirmed for mature PECs, including paired box gene 2 (PAX2), paired box gene 8 (PAX8), Claudin-1, Claudin-2, and Claudin-16 (Ohse et al., 2009a,b). Interestingly, most recent studies have suggested that PECs are directly involved in the pathogenesis of certain glomerular diseases, which are characterized by the activation of specific progenitor cell markers of PECs, including CD44, CD24, CD133, phosphorylated extracellular signal-regulated kinase, and several other molecules (Ohse et al., 2009a; Eng et al., 2015). CD24 and CD133 are also used to identify human stem or progenitor cells in several adult human tissues (Dziedzic et al., 2014). The biological properties of activated PECs mainly present as increased proliferation and migration (Ohse et al., 2009b). Activated PECs play a potential role in glomerular repair by partially or completely rescuing the reduction in the number of podocytes, serving as progenitor cells for podocytes (Zhang et al., 2013).

During nephrogenesis, podocytes and PECs are developed from the metanephric mesenchyme, which is induced by the ureteric bud to generate S-shaped bodies. Further, elongation occurs as the middle and distal segments of the S-shaped body are in contact with the ureteric bud epithelium to form distal renal tubular. The proximal end is invaded by blood vessels, and Bowman’s space begins to form. Between the S-shaped body and capillary loop stages, PECs and podocytes begin to express unique marker proteins specific to each cell’s function. Both the shared common lineage between PECs and podocytes and their close connection makes PECs excellent potential candidates as podocyte precursors (Ohse et al., 2010). Some previous encouraging findings proved that activated PECs can express marker proteins considered specific for podocytes. Schulte and colleagues used lineage tracing to show that CD133- and CD24-positive PECs co-express the podocyte marker synaptopodin in human kidneys (Schulte et al., 2014). Similarly, Lasagni et al. provided evidence that adult human glomeruli contain a population of stem and committed progenitor cells localized within the Bowman’s capsule; these cells are characterized by the co-expression of two surface markers, CD24 and CD133, and exhibit self-renewal properties as well as the potential to differentiate into podocytes (Lasagni and Romagnani, 2010). Furthermore, in a seminal study by Appel et al. (2009), they performed Cre recombination in triple-transgenic pPECrtTA/LC1/R26R adolescence mice using labeled β-gal-positive PECs, which migrated to the glomerular tuft and co-expressed the podocyte marker proteins nephrin, synaptopodin, and WT-1, and fully differentiated into podocytes. Under cell culture conditions, Kietzmann et al. (2015) supplemented PECs with vitamin D3, retinoic acid, and dexamethasone (VRADD) to induce their differentiation into podocytes (Dai et al., 2017). This transformation link between PECs and podocytes in the repair and replenishment of decreased podocyte number within the glomerular tuft provides an important basis for a thorough understanding of the mechanism and the pathogenesis of podocytopathies.

These recent studies showed that PECs may serve as a potential precursor for podocytes and play an important role in various podocytopathies. This review summarizes a significant role of PECs in the development and progression of podocytopathy.

## Parietal Epithelial Cells in Kidney Physiology

**FIGURE 1:**
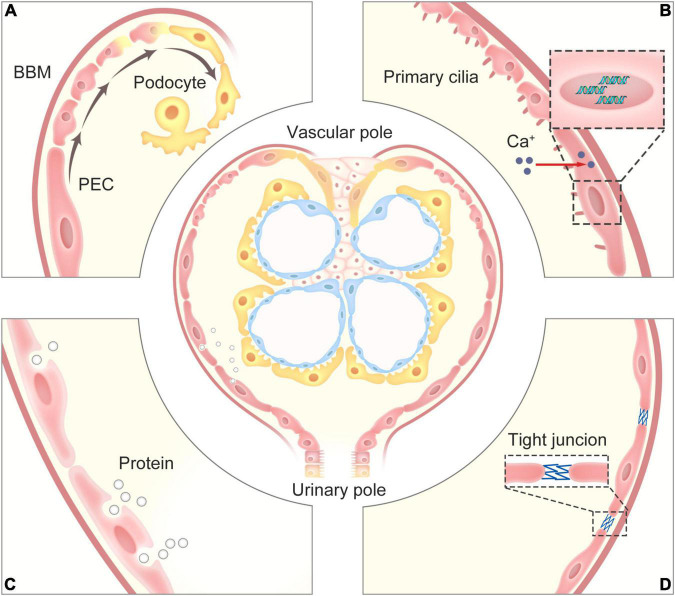
The physiological functions of PECs. **(A)** PECs serve as a potential precursor for podocytes, which can progressively proliferate and differentiate into podocytes for restore and maintain the number of podocyte within the glomerular tuft. **(B)** The primary cilia on the surface of PECs can perceive the change of flow from the glomerular filtrate to promote the increase in intracellular calcium that affects gene expression, and the contractility of PECs may regulate the pressure in the Bowman’s capsule to adjust glomerular filtration. **(C)** PECs can take up proteins *via* an undefined endocytosis mechanism in cases of glomerular ultrafiltrate overload. **(D)** PECs with their tight junctions form an impermeable barrier, prevents proteins in the glomerular ultrafiltrate in the Bowman’s space from exiting into the extraglomerular space. PECs, parietal epithelial cells; BBM, Bowman’s basement membrane.

Compared to other kidney resident cells, there are few universally accepted concepts about the physiological functions of PECs, some of them are still in the stage of discussion. We have collected the available evidence indicating their functions (Figure 1).

### Role in Mechanosensation and Contractility

Similar to renal tubular epithelial cells, the structure of primary cilia on the surface of PECs may play an important role in chemical and mechanical sensation (Yoder, 2007). There is speculation that primary cilia are responsible for perceiving the change of flow from the glomerular filtrate to promote the increase in intracellular calcium that affects gene expression (Ohse et al., 2009b). Further experiments confirmed that PECs have contractility. Webber and Wong (1973) divided the experimental group into further three groups depending on whether the rats were injected with histamine, acetylcholine, or epinephrine. The common carotid artery was infused with fixative fluid, and the kidneys were separated and sectioned. The control group was directly infused with a fixative solution. Electron microscopy showed that the membrane of PECs in rats in the epinephrine group was slightly folded, while the lateral and the basement membranes of PECs in the control group were smooth, indicating that PECs have a contractile function (Webber and Wong, 1973). Thus, we hypothesized that PECs may adjust glomerular filtration by regulating the pressure in the Bowman’s capsule through a contractile mechanism, but further convincing evidences are required.

### Role in the Glomerular Barrier and Protein Uptake

As PECs are attached to BBM, tracer studies were performed by Kriz et al. (2001) to suggest that PECs together with BBM constitute a barrier, which prevents misdirected glomerular filtrate and peritubular filtrate from spreading into extracellular spaces. Ohse et al. (2009a) further determined that tight junctions (TJs) are present between adjacent PECs in normal glomeruli. This important function of PECs, through the expression of TJ proteins claudin-1, zonula occludens-1 (ZO-1) and occludin to form an impermeable barrier, prevents proteins in the glomerular ultrafiltrate in the Bowman’s space from exiting into the extraglomerular space (Ohse et al., 2009a). However, under disease conditions, the decrease in the number of TJ proteins is associated with the increased distances between PECs, allowing the glomerular ultrafiltrate to pass through the detached barrier into the extraglomerular space. It is speculated that PECs take up proteins in cases of glomerular ultrafiltrate overload *via* an undefined endocytosis mechanism, causing PEC injury.

### Role as a Podocyte Progenitor

As mentioned above, PECs and podocytes are derived from common ancestral mesenchymal cells. Unlike podocytes, PECs maintain the ability to proliferate. Some researchers have thought that PECs may serve as a potential precursor for podocytes. Indeed, even under normal physiological conditions, detached podocytes can be found in the urine. Hence, cell regeneration and repair is necessary to replace the lost podocytes. In a study of aging nephropathy, Zhang et al. (2012a) found that podocyte number decreases with aging while the number of PECs increases, accompanied by an increase in the number of glomerular transition cells expressing specific proteins for PECs and podocytes. This raises a possibility that PEC proliferation with aging happens to compensate for their transitioning to restore and maintain the podocyte number (Kaverina et al., 2020). The potential of PECs to effectively differentiate into podocytes may reduce undergrowth stress (Schneider et al., 2017). As early, Gibson et al. (1992) described the presence of parietal cells at the vascular pole in the human kidney, which resemble visceral podocytes lining the inner membrane of the Bowman’s capsule. Accordingly, these parietal cells are defined as parietal podocytes (pPods). Bariety et al. (2006) further confirmed that pPods exist in the normal human Bowman’s capsule, co-expressing PEC and podocyte-specific proteins (Bariety et al., 2006). Whether pPods are derived from the transdifferentiation of PECs to compensate for the loss of podocytes was not fully proven until recently. Kaverina et al. (2020) found that a subpopulation of PECs replace podocytes during ageing *via* lineage tracing using a dual PEC-Podocyte double fluorescent reporter mouse model. This study indicated that PECs driving podocyte replacement express podocyte markers podocin, nephrin, and p57, and acquire ultrastructural features of podocytes. Although they still express the activation marker for PECs, CD44, they no longer express the PEC marker PAX8 and lose the capacity to proliferate. Moreover, podocytes derived from the PEC subpopulation express vascular endothelial growth factor-A (VEGF-A), the key factor maintaining the structure and function of the glomerular capillary (Kaverina et al., 2020). Zhang et al. (2012b) recently discovered that alltrans retinoic acid (ATRA) increases the number of glomerular epithelial transition cells and podocytes in proteinuric glomerular diseases, suggesting that ATRA may provide a useful pharmacological approach to explain the mechanisms underlying the possible progenitor role of PECs, and transplantation of mesenchymal stem cells preserves the potential of PECs *via* modulating their activation (Aslam et al., 2020).

## Signaling Pathways Regulate Parietal Epithelial Cell Activation, Proliferation, and Pro-Fibrogenic Effects

**FIGURE 2:**
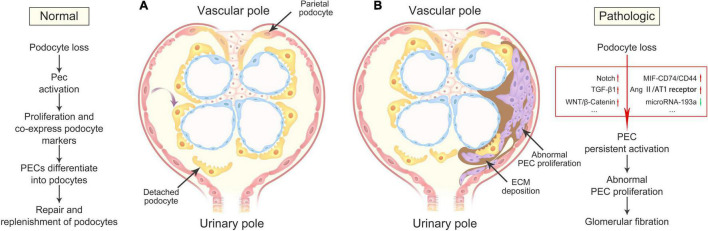
Outcomes of PEC activation in health and disease. **(A)** Under physiologic conditions, damage or loss of podocytes leads to PEC activation, resulting in its progressively proliferation and differentiation into podocytes, and consequently repair and replenishment of differentiated podocytes lost during normal nephron function. **(B)** Under pathologic conditions, severe podocyte death or detachment contributes to the abnormalities of multiple signaling pathways, including Notch, TGF-β1, WNT/β-Catenin, MIF-CD74/CD44, Ang II/AT1 receptor, and microRNA-193a, resulting in the accumulations of abnormal proliferating PEC and ECM in the Bowman’s space, and finally glomerular fibration. PECs, parietal epithelial cells; ECM, extracellular matrix.

The signaling pathways that regulate PEC activation, proliferation, and pro-fibrogenic effects are just partially comprehended, however, with the in-depth understanding of PECs, more and more potential signaling pathways will be revealed. Getting a closer look at them will offer us practical clues for the pathogenesis and clinical treatment of disease. We summarize the major signaling pathways related to PEC activation, proliferation, and pro-fibrogenic effects in Figure 2.

### Notch-Dependent Parietal Epithelial Cell Activation and Epithelial-Mesenchymal Transition Mediated by Transforming Growth Factor-β1

Notch is a single-transmembrane protein, which plays a critical role in kidney development (Sharma et al., 2011). The Notch signaling pathway is an evolutionarily conserved cell-cell communication mechanism composed of at least four Notch receptors (Notch 1–4) and five Notch ligands (Delta-like 1, 3, 4, and Jagged 1–2), which is involved in cell migration, proliferation, and phenotypic transformation in many diseases (Talora et al., 2008). Generally, the activity of the Notch signaling pathway significantly decreases as the glomerular development is completed (Sharma et al., 2011). Elevated expression of Notch proteins, as well as the related ligands, has been confirmed in different kinds of podocytopathies, including FSGS and DN. Activated Notch signaling was also reported to correlate with the development of tubulointerstitial fibrosis (Edeling et al., 2016). For glomerular epithelial cells, increased Notch expression in podocytes correlated with albuminuria and glomerulosclerosis in some podocytopathies (Niranjan et al., 2008). In recent years, compelling findings have been published to support a critical role for the Notch signaling pathway in PEC activation in focal segmental glomerulosclerosis (FSGS) and diabetic nephropathy (DN). A confirmatory experiment was performed by Ueno et al. which indicated increased expression of Notch signaling-related proteins (Notch1, Jagged1, and Delta1), reflecting Notch signaling activation, in PECs of a collapsing FSGS transgenic mouse model and human collapsing FSGS (Ueno et al., 2013). In addition, CD24- and CD133-positive PECs in patients with FSGS also upregulated Notch3 expression levels (Lasagni et al., 2010). As mentioned above, activated PECs can migrate from Bowman’s capsule to the affected segment of the glomerular tuft; however, cell migration is reduced after inhibition of Notch signaling activity (Ueno et al., 2013). These results demonstrate that Notch signaling is one of the regulatory mechanisms in PEC activation and migration.

Epithelial–mesenchymal transition (EMT) describes the process of epithelial cells losing their epithelial characteristics and transforming into mesenchymal cells. Partial EMT in renal epithelial cells plays a significant role in the occurrence and development of interstitial fibrosis. Notch is a strong regulator of the main transcription factors involved in EMT; this process is likely to be important in replacing lost epithelial cells following injury (Barak et al., 2012; Sirin and Susztak, 2012). Expression of Notch receptors was observed in tubular epithelial cells, which can drive transdifferentiation of epithelial cells into activated myofibroblasts (Bielesz et al., 2010). However, whether this transition occurs in PECs is still unknown. To further explore if Notch-dependent EMT contributes to the development of fibrosis in PECs during FSGS, Ueno and colleagues treated cultured PECs with Transforming Growth Factor-β1 (TGF-β1), a multifunctional mediator that can stimulate Notch and myofibroblast activation, which resulted in a significantly higher expression of mesenchymal cell phenotype markers (Ueno et al., 2013). Preincubation with dibenzazepine (DBZ), a γ-secretase inhibitor, can block EMT production mediated by TGF-β1 in PECs. These mesenchymal marker proteins, including α-smooth muscle actin (αSMA) and vimentin, cause the deposition of extracellular matrix (ECM), which may be a pathological base for aggravating the formation of glomerulosclerosis (Ueno et al., 2013). Furthermore, studies have demonstrated that Smad7 overexpression can inhibit inflammation and renal fibrosis by blocking the activation of the TGF-β/Smad signaling pathway (Lan et al., 2003; Chen et al., 2011). This protective role of Smad7 may present new therapeutic potential for treating glomerulosclerosis caused by TGF-β1 activation in PECs.

Consequently, the pro-fibrogenic effects of the Notch signaling pathway in PECs in response to podocyte loss may mediate PEC activation, migration, and mesenchymal phenotypic alteration *via* TGF-β1.

### Wnt/β-Catenin Signaling Pathways in Parietal Epithelial Cell Activation and Fibrosis Development

The Wnt/β-Catenin signaling pathway is a highly conserved pathway that is mainly involved in cell proliferation and differentiation, inflammation, and fibrosis. In addition, this multifunctional pathway plays a key role in kidney development and glomerular diseases (Grouls et al., 2012).

Recent reports suggest that the Wnt/β-Catenin signaling pathway is reactivated in response to glomeruli injury associated with regeneration and repair. A previous study showed that Wnt/β-Catenin signaling induces podocyte injury and proteinuria *via* TGF-β1 (Wang et al., 2011). Later studies performed by Kato and colleagues indicated that increased Wnt/β-Catenin signaling negatively regulates podocyte differentiation markers and promotes PEC-specific marker expression (Kato and Susztak, 2012). Explicit data revealed that Wnt/β-catenin signaling also modulates the development of fibrosis (Edeling et al., 2016). Whether Wnt/β-catenin signaling-induced fibration occurs in PECs after glomerular damage remains to be investigated. More importantly, TGF-β1 can activate Wnt/β-catenin and concomitantly suppress α-Klotho expression. Loss of α-Klotho promotes fibration *via* the activation of Wnt/b-Catenin, suggesting that α-Klotho could be a rational strategy to attenuate renal fibrosis for inhibiting pathogenic Wnt/β-catenin signaling in activated PECs (Zhou et al., 2013).

### Macrophage Migration Inhibitory Factor Activates CD74 and CD44, Leading to Pathological Parietal Epithelial Cell Proliferation and Crescent Lesion Formation

Macrophage migration inhibitory factor (MIF), a pleiotropic, proinflammatory cytokine, produced by injured podocytes (Ito et al., 2020), is a signaling molecule that mediates pathological PEC proliferation *via* CD74/CD44, thereby leading to an aggressive and progressive course of crescentic glomerulonephritis. CD74 is a cell-surface transmembrane glycoprotein that provides a binding site for MIF, and functions intracellularly as an MHC class II chaperone (Djudjaj et al., 2016). In addition to CD74, the signal transduction of MIF requires the recruitment and activation of CD44, a PEC activation marker, to induce PEC activation and pathological proliferation in crescentic glomerulonephritis (Djudjaj et al., 2016). The formation of cellular crescents as a result of PEC activation and proliferation, which plays a vital role in the development and progression of crescentic glomerulonephritides, is now becoming increasingly clear. As markers for PEC activation, upregulation of both CD74 and CD44 was verified in an FSGS mouse model (Yamazaki et al., 2016). Another credible piece of evidence was reported by Djudjaj et al. (2016) that MIF, as well as its receptor complex CD74/CD44, is upregulated in the glomeruli of patients and mice with proliferative glomerulonephritides. However, genetic MIF/CD74 deficiency or blocking MIF activity can ameliorate glomerular injury and partially reverse established crescentic glomerulonephritis (Yang et al., 1998; Djudjaj et al., 2016). Phosphorylation of extracellular signal-regulated kinase (p-ERK) may be the key molecule induced by the CD44/CD74 receptor complex triggered by MIF (Leng et al., 2003; Hamatani et al., 2020). Moreover, increased albumin levels in initial urine induce CD44 expression in PECs *via* activating the megalin-mediated ERK signaling pathway (Zhao et al., 2019). Activated ERK1/2 increases CD44 expression in glomerular PECs, leading to the production of more ECM, which forms a positive feedback loop to promote glomerular scarring (Roeder et al., 2017).

Furthermore, interactive activation between MIF and T cells is a pivotal mechanism in regulating the inflammatory and immune responses in several kidney diseases tightly associated with the glomerular crescent formation. MIF, once activated, can induce the production of other inflammatory cytokines and initiate the inflammatory cascade, further promoting kidney injury (Lan, 2008).

Therefore, MIF-CD74/CD44 signaling pathway likely implicates a potential mechanism for PECs in the pathological proliferation, as well as crescent lesions formation. Of note, ribosomal protein S19 (RPS19), identified as the first endogenous MIF inhibitor that blocks the binding of MIF to its receptor CD74, may be a potent anti-inflammatory agent that prevents the development of glomerular crescents induced by PECs (Lv et al., 2013).

### Other Pathways Leading to Parietal Epithelial Cell Activation and Fibrosis Development

Besides the three main signaling pathways introduced above, several other signaling pathways affecting PEC activation and fibrosis development have also been described, including heparin-binding epidermal growth factor-like growth factor (HB-EGF)/EGFR (Flamant et al., 2012), CXC chemokine receptor-4 (CXCR4)/stromal cell-derived factor-1 (SDF-1) (Rizzo et al., 2013), amino acid transporter (LAT)/mTORC1 (Kurayama et al., 2011), MAP-Kinase (Eng et al., 2015), mitotic spindle assembly checkpoint protein 2 (MAD2B)/S phase kinase-associated protein 2 (Skp2) (Ye et al., 2020), and angiotensin II (Ang II)/type-1 (AT1) receptor (Benigni et al., 2011; Rizzo et al., 2017) signaling pathways. Furthermore, overdose of ACE inhibitors, retinoids, and vitamin D in some diseased animal models reportedly enhanced the progenitor capacity of PECs (Benigni et al., 2011; Wang et al., 2012; Zhang et al., 2012b).

Regulation of gene expression might provide another available means for PEC activation. Recent studies indicated that microRNA-193a inhibition in human PECs may mediate the transition of PEC to podocyte phenotype, accompanied by preclusion of proliferation, migration, and crescent formation (Kietzmann et al., 2015; Lazzeri and Romagnani, 2015). Kumar et al. (2018) also showed that apolipoprotein L1 (APOL1) regulates PEC molecular phenotype through modulating microR193a expression.

Recently, CD9 has been proven to promote the migration of PECs into the glomerular tuft, where they acquire CD44 and β1 integrin expression (Lazareth et al., 2019; Smeets et al., 2020). However, these potential candidate pathways require further convincing evidence.

## Parietal Epithelial Cells in Podocytopathy

Podocytopathy is the most common feature of glomerular disorders, mainly characterized by podocyte injury, dysfunction, or loss, leading to a broad spectrum of clinical syndromes. FPs and the deposition of immunopathologic oligoimmune complexes can be observed under an electron microscope, while under a light microscope, podocytopathy shows diversity, such as mild mesangial hyperplasia, podocyte swelling, and podocyte proliferation. Based on the pathophysiology, light microscopy, and ultrastructural evaluation, podocytopathies can be divided into several pathological patterns. Among them, minimal change disease (MCD), focal segmental glomerulosclerosis (FSGS), membranous nephropathy (MN), diabetic nephropathy (DN), IgA nephropathy (IgAN), and lupus podocytopathy are the relatively common types observed in the clinic. The progression of podocytopathy is a complicated series of events regulated by multiple factors. Toxic, medical, genetic, immune, infectious, oxidant, metabolic, and other mechanisms can all target podocytes. Apart from MCD, unfortunately, a significant number of other common types have a poor prognosis and finally progress to end-stage renal disease (ESRD). It is well known that podocyte depletion is strongly correlated with progressive glomerular disease in humans and is a driver of glomerular disease progression in animal models (Kopp, 2015). It is suggested that loss of podocytes beyond a certain threshold is sufficient to trigger glomerulosclerosis, which then develops into ESRD (Schulte et al., 2014). Therefore, targeting the repair and replenishment of podocytes within the glomerular tuft is a key point for the treatment of podocytopathies. PECs may play a significant role in this process, according to a recent report. These findings are summarized in Table 1.

**TABLE 1:** Relevant information on PECs activation in podocytopathy.

		References	
		
	Signaling pathways (or key molecules)	Human	Animal model
MCD	Claudin-1, CD44, LKIV69	Smeets et al., 2014	
	CD44	Fatima et al., 2012	
	PAX8	Suzuki et al., 2020	
FSGS	Notch	Lasagni et al., 2010 Ueno et al., 2013	Lasagni et al., 2010
			Ueno et al., 2013
	TGF-β1, EMT	Ueno et al., 2013	Ueno et al., 2013
	Wnt/β-Catenin		Kato and Susztak, 2012
	MIF-CD74/CD44	Djudjaj et al., 2016	Djudjaj et al., 2016
			Yamazaki et al., 2016
	ERK	Roeder et al., 2017	Roeder et al., 2017
	KLF4/STAT3		Estrada et al., 2018
			Pace et al., 2021
	Claudin-1, WT-1, PAX8,		Ohse et al., 2010
	CD44, synaptopodin.		Eymael et al., 2018
	Podocin, nephrin, p57		Kaverina et al., 2019
	CD44, p57, podocin		Eng et al., 2015
	CD44, LKIV69	Smeets et al., 2011	Smeets et al., 2011
MN	Claudin-1, WT-1, PAX8,		Ohse et al., 2010
	CD44, synaptopodin.		
	WT-1, PAX2	Bariety et al., 2006	
DN	Notch	Lasagni et al., 2010	Lasagni et al., 2010
	ERK		Zhao et al., 2019
	Claudin-1, WT-1, p57	Andeen et al., 2015	Pichaiwong et al., 2013
			Andeen et al., 2015
	Gas1, PAX2, WT-1		Luna-Antonio et al., 2017
	MIF-CD74/CD44	Chan et al., 2019	Chan et al., 2019
	CD44, TGF-β1	Holderied et al., 2015	
IgAN	CD44, PAX8, WT-1	Kim et al., 2016	
	PAX2, WT-1	Florquin et al., 2002	
		Hill et al., 2011	
LN	CD44, LKIV69	Weening et al., 2004	
		Kuppe et al., 2015	

*MCD, minimal change disease; FSGS, focal segmental glomerulosclerosis; MN, membranous nephropathy; DN, diabetic nephropathy; IgAN, IgA nephropathy; LN, lupus nephritis; LKIV69, PEC matrix; PAX8, paired box gene 8; TGF-β1, transforming growth factor-β1; EMT, epithelial-mesenchymal transition; MIF, macrophage migration inhibitory factor; ERK, extracellular signal-regulated kinase; KLF4, Krüppel-like factor 4; STAT3, signal transducer and activator of transcription 3; WT-1, wilms tumor protein 1; PAX2, paired box gene 2; Gas1, growth arrest-specific 1.*

### Minimal Change Disease

Minimal change disease (MCD) and FSGS are the two forms of acquired glomerular disease that are considered to be the most characteristic podocytopathies, but in contrast to FSGS, glomerular sclerosis lesions are absent in MCD. The pathogenesis of MCD, the most common and simplest form of podocytopathies in children, which also occur in young adults, has not yet been fully elucidated. MCD is characterized by extensive podocyte FP effacement observed under an electron microscope but no abnormalities in the glomeruli are observed using light microscopy. These ultrastructural changes in podocytes may be responsible for the increased glomerular permeability and proteinuria. It is obvious that MCD differs from other podocyte-related diseases, without podocyte depletion. However, some experts have considered that MCD potentially progresses to FSGS, representing different manifestations of one disease. Others have suggested that even in the early presclerotic stage, FSGS is distinct from MCD. Nevertheless, the relationship between MCD and FSGS has remained controversial (Garin et al., 2010). Notably, small lesions particularly at the glomerular tip may be missed in biopsies initially diagnosed as MCD (Smeets et al., 2014). The recent discovery of activated PECs can help to distinguish early FSGS from MCD (Smeets et al., 2014). As previously reported, there is an increased number of PECs within the glomerular tuft that expresses the activation marker CD44 in the early stages of FSGS, whereas few activated PECs are observed in MCD (Fatima et al., 2012). A further study performed by Smeets et al. described three novel markers, PEC matrix (LKIV69), A-kinase anchor protein 12 (AKAP12), and Annexin A3 (ANXA3), expressed by PECs in early FSGS that can be used to improve the sensitivity for distinguishing early FSGS from MCD (Smeets et al., 2014).

Detection of PEC markers may be valuable for understanding the pathogenesis of MCD, particularly for diagnostic purposes to distinguish early FSGS from MCD (Suzuki et al., 2020), although the biological consequence of PEC activation markers linking MCD and FSGS is unclear.

### Focal Segmental Glomerulosclerosis

Focal segmental glomerulosclerosis (FSGS), the most common primary glomerulopathy causing ESRD, is characterized by focal and segmental obliteration of glomerular capillary tufts with a thicker matrix. According to the location and character of the sclerotic lesion, FSGS is classified as collapsing, tip, cellular, perihilar, and not otherwise specified variants. Central to the pathogenesis of FSGS, the most common cause of nephrotic syndrome in the US is damage to podocytes, resulting in their loss. It is well known that when the number of podocytes is below a critical threshold, glomerulosclerosis ensues (Kim et al., 2001; Wharram et al., 2005). However, podocytes are terminally differentiated epithelial cells that are typically unable to adequately proliferate. Thus, attempting to identify possible progenitors that might replace podocytes is the main treatment strategy for FSGS (Nishizono et al., 2017). In recent studies, it has been suggested that PECs are attractive candidates to serve as podocyte progenitors (Ohse et al., 2010; Eng et al., 2015). Compared to other podocytopathies, the research on the mechanism of how PECs differentiate into podocytes is mainly concentrated on FSGS. In FSGS models, PECs can transdifferentiate into adult podocytes expressing podocyte markers podocin, nephrin, p57, and VEGF164 (Kaverina et al., 2019). Eng and colleagues showed that a subset of PECs on the glomerular tuft co-express the activation marker CD44 in a mouse model of FSGS, and that these activated PECs migrate from Bowman’s capsule to the glomerular tuft to become podocyte progenitors, as this regeneration contributes to an increase in the number of podocytes, which is accompanied by decreased scarring (Eng et al., 2015). Similar findings were reported in another study reporting that an increasing number of PECs co-express PAX8 and synaptopodin within the glomerular tuft in the adriamycin (ADR)-induced mouse model of FSGS (Ohse et al., 2010). Besides CD44 and CD74 can also serve as a marker for PEC activation in FSGS. By modifying the ADR mouse model using LPS treatment, Yamazaki et al. (2016) found that CD74 upregulation better reflects a rapid amplification of PEC activation than CD44 expression.

From the above studies, we can deduce the capacity of mature PECs to proliferate and differentiate into podocytes, which likely play a role in the repair and replenishment of decreased podocyte numbers within the glomerular tuft. However, a considerable amount of studies disclosed that activated PECs are involved in the pathogenesis and progression of glomerulosclerosis. To determine whether PECs contribute to sclerosis in the progression of FSGS, Moeller and colleagues provided strong evidence that focal activation of PECs leads to cellular adhesions between Bowman’s capsule and the capillary tuft. The adhesion then provides an entry site through which PECs migrate into the capillary tuft and eventually lead to glomerulosclerosis. Three distinct models of FSGS (5/6-nephrectomy DOCA-salt model; murine transgenic chronic Thy1.1 model; MWF rat model) and human biopsies were used to observe this phenomenon (Smeets et al., 2011). Similarly, results observed in crescentic glomerulonephritis and collapsing glomerulopathy proved that PECs can proliferate to form cellular glomerular lesions (Smeets et al., 2011; Wong et al., 2021). The findings reported by Bariéty et al. (2001) are in agreement with the above studies and further illustrate that after transdifferentiation, PECs migrate into podocyte-depleted areas in the tuft and that primary FSGS as well as recurrence after primary transplantation is followed by sclerosis formation. In addition, Eymael et al. (2018) observed that CD44 deficiency results in decreased glomerular cell proliferation and reduced albuminuria, which revealed that acquired glomerular CD44 expression by activated PECs is required for the pathogenesis of crescentic glomerulonephritis and collapsing FSGS (Eymael et al., 2018). A retrospective study performed by Froes et al. (2017) indicated, for the first time, that CD44 positivity in PECs can be a pathological marker for renal function deterioration in pediatric patients with FSGS. In collapsing FSGS, the reduced expression of Krüppel-like factor 4 (KLF4) in podocytes triggered IL-6 release into the supernatant, which stimulated PEC activation *via* signal transducer and activator of transcription 3 (STAT3) pathway (Estrada et al., 2018). By using RNA-sequencing, mass spectrometry, and single-cell RNA-sequencing, Pace et al. have identified key ligand-receptor interactions between podocytes and PECs *via* KLF4/STAT3 signaling. Restoration of KLF4 expression in podocytes or inhibition of STAT3 signaling prevented the loss of podocytes and suppressed PEC activation in mice (Pace et al., 2021).

However, it is still not clear how PECs respond to replenish the decreased number of podocytes. The validity of these findings remains to be determined. The most likely interpretation is that PECs, cannot supersede the physiological function of podocytes. Once on the glomerular tuft, PECs are incapable of secreting adequate vascular endothelial growth factor, and this triggers endothelial problems, including collapse and scarring in the affected capillary (Hakroush et al., 2014). However, this view has recently been challenged (Kaverina et al., 2020). Furthermore, migrated PECs may induce inflammatory cytokine expression, which activates an inflammatory response, leading to capsular synechia or even fibration. Considering the indispensable role of PECs in the progression of FSGS, more related research about signaling pathways that mediate sclerosis formation are emerging. The major signaling pathways are mentioned above.

In addition, PECs may also be activated in the absence of a primary podocyte injury. In a mouse model of hypertensive nephropathy, endothelial-specific deletion of PAS domain-containing protein 1 promotes the activation of PECs and accelerates FSGS induced by angiotensin II. Thus, endothelial injury can also trigger FSGS *via* activating PECs (Luque et al., 2017).

### Membranous Nephropathy

Membranous nephropathy (MN) is a common cause of podocytopathies in adults, characterized by complement activation-induced podocyte sublethal damage and even detachment from the glomerular tuft. In this pathological process, the immune reaction is the initiation factor and podocyte injuries are crucial events in the development of severe proteinuria. Emerging evidence indicates that PEC activation accompanied with pseudocrescent formation is sometimes observed in MN (Ohse et al., 2010; Barrett et al., 2014). Whether PEC activation triggered by podocyte injury plays a role in the pathogenesis of MN has attracted increasing attention recently.

In the passive Heymann nephritis (PHN) rat model of MN, the data presented by Ohse et al. (2010) show that double-positive cells for PEC and podocyte marker proteins are present in the glomerular tuft, as well as along the BBM. One of the explanations is that PECs as progenitor cells differentiate into podocytes to repair and replenish decreased podocyte numbers within the glomerular tuft (Ohse et al., 2010). However, in membranous glomerulopathy in humans, PAX2-positive PECs were observed using transmission electron microscopy between the GBM and the BBM; this structure is defined as a podocyte bridge that initiates the formation of crescents (Bariety et al., 2006). The mechanisms that regulate these events need to be further explored.

### Diabetic Nephropathy

Diabetic nephropathy (DN), one of the serious complications of diabetes mellitus, is a leading cause of the progressive decline in kidney function leading to ESRD. As a typical secondary podocytopathy, podocyte injury and loss cause the aggravation of DN and even its progression toward glomerulosclerosis. Thus, reversal of the structural and functional abnormalities of DN requires the restoration of the number of podocytes. Recent studies found that PECs may serve as progenitor cells and may play a potential role in the restoration of podocyte numbers in DN (Andeen et al., 2015). These findings provide some evidence for phenotypic plasticity in PECs and podocytes, by which activated PECs may proliferate and co-express podocyte markers, and then migrate to promote podocyte restoration or ameliorate podocyte loss. A similar result was obtained using a BTBR ob/ob diabetic mouse model by Pichaiwong and colleagues Pichaiwong et al. (2013), where they significantly reversed proteinuria and other morphological abnormalities, suggesting a reparative role for activated PECs. Furthermore, Luna-Antonio et al. (2017) suggested that the loss of growth arrest-specific 1 (Gas1), a pleiotropic protein with novel functions including anti-proliferative and proapoptotic activities, in renal damage due to diabetes promotes the activation of parietal progenitor cells in the Bowman’s capsule that might differentiate into podocytes and compensate for their loss. Inconsistently, the number of activated PECs were found to be correlated with podocyte loss and proteinuria (Zhao et al., 2011), as well as thickening of Bowman’s capsules and ECM production in diabetic glomerulosclerosis (Holderied et al., 2015). Consequently, the potential effect of activated PECs on the pathogenesis of DN remains controversial.

The mechanism of PEC activation in DN remains unclear. Several signaling pathways related to activated PECs involved in the pathogenesis of DN have been reported. Majority of these focused on Notch (Lasagni et al., 2010; Edeling et al., 2016), Wnt/β-Catenin (Kato and Susztak, 2012), MIF-CD74/CD44 (Bruchfeld et al., 2016; Djudjaj et al., 2016; Chan et al., 2019), and LAT/mTORC1 signaling pathways (Kurayama et al., 2011), as mentioned above.

### IgA Nephropathy

IgA nephropathy (IgAN), which is characterized by a broad spectrum of clinical presentations and pathologic findings, is the most common form of primary glomerulonephritis worldwide. About 50% of patients with IgAN will progressively develop ESRD within 30 years despite treatment (Moriyama et al., 2014). It is known that lesions morphologically identical with FSGS may appear in IgAN. Hill et al. (2011) strongly suggested that podocytopathy, similar to that observed in primary FSGS, occur frequently in IgAN. They found that podocyte and PEC alterations observed in IgAN are identical to those observed in primary FSGS, which are related to the progression of the disease. Changes in podocytes begin focally, which was not previously realized, and with the progression of lesions, podocytes are progressively lost and replaced by PECs. Interestingly, CD44 expression was observed in PECs and podocytes in IgAN (Kim et al., 2016). Among these patients, increased CD44 expression in podocytes is a sign of active glomerular injury and dysfunction, and both CD44-positive PECs and podocytes are related to segmental sclerosis or synechia in IgAN. Moreover, the expression of CD44 correlates not only with the degree of renal damage but also with proteinuria in adults with IgAN (Kim et al., 2016), and could be a reliable marker of the progression of IgAN (Florquin et al., 2002).

### Lupus Podocytopathy

Lupus podocytopathy, characterized by diffuse FP effacement without peripheral capillary wall immune deposits and glomerular proliferation, has been described in patients with systemic lupus erythematosus with nephrotic syndrome over the last 20 years (Nishihara et al., 1997; Hertig et al., 2002; Wang et al., 2006; Hu et al., 2016). It might be a distinct class of lupus nephritis (LN) and this class of LN is not part of the current classification of LN (Weening et al., 2004; Gutiérrez et al., 2012). There are few studies on whether PECs are involved in the pathogenesis of lupus podocytopathy. Kuppe et al. (2015) have identified PEC markers in virtually all secondary FSGS lesions, including LN. In LN, infiltrating immune cells were detected at a higher frequency, but cells expressing PEC markers still comprised the majority of cells in cellular lesions. Furthermore, cellular crescents are a feature of active LN (Weening et al., 2004). Cellular crescents commonly overlie necrosis of the glomerular tuft and are formed by proliferating PECs with infiltrating mononuclear cells (monocytes or macrophages).

## Perspectives and Conclusion

Although there is currently no data to support the association of a disease with primary PEC lesions, PECs are active in a variety of glomerular diseases, such as podocytopathies. The interaction between PECs and podocytopathies is complex and important. Understanding the changes in the structure and function of the physiology and pathological states helps us better understand the occurrence and development of various podocytopathies, and further explore possible therapeutic targets. PECs not only play a significant role in mechanosensation, contractility, glomerular barrier, and protein uptake but it also seems likely that activated PECs serve as progenitor cells to replenish the decreased number of podocytes in podocytopathies, which provides an exciting idea to reverse the progression of human glomerular diseases. Nonetheless, the underlying signaling pathways of how activated PECs are involved in reparative or injurious effects remain controversial. We need to further explore how these signaling pathways or molecular targets are regulated to hasten repair and reduce the progression of podocytopathies. For further exploration, the patterns of initiation within glomerular fibration triggered by activated PECs should be studied. It is now clear that podocyte injury is the initial event of PEC activation; however, detailed machinery of signal transduction remains unknown. There is space between the glomerular tuft and the Bowman’s capsule which might indicate a pattern of extracellular signal transduction to control the target cell. It was found that TGF-β1-containing exosomes secreted by injured epithelial cells into the blood and extracellular fluid have roles in kidney regeneration and fibrosis (Borges et al., 2013). Whether this potential exosome-mediated initial signaling activates PECs induced by injured podocytes requires further confirmation. Moreover, studies mentioned above demonstrate repair and replenishment effects of activated PECs on podocyte loss as a useful initial process, but persistent activation of PECs finally promote glomerular fibration, though the exact signaling pathway still unclear, which is a harmful outcome (Figure 2). Does the defective termination signal for PEC activation contribute to fibration? If so, how does this process occur? In addition, PECs include three subpopulations as follows: Classical flat PECs, cuboidal PECs, and intermediate PECs. Cuboidal and intermediate PECs are activated easier than the classical flat subgroup and form glomerular sclerotic lesions and intermediate PECs are especially involved in tip lesions (Kuppe et al., 2019). The existing research is still rare in this field.

The last decade has seen a significant increase in our understanding of PEC activation and transdifferentiation for the pathophysiological processes underlying kidney development and disease. While there is a long way to go in understanding the roles of PECs play and the signaling pathways involved during response to kidney injury, especially in various podocytopathies, our increasing attention to the physiological function of PECs and its lineage relationships with podocyte will continue to inform us about the pathogenesis of glomerulopathies. The hope is that this will ultimately instruct the development of therapeutic approaches to improve the outcome for the patient.

In this review, we summarized current literature related to the pathophysiology of PECs in the pathogenesis of podocytopathies. We majorly focused on narrating the double-effect of PECs, either reparative or injurious, activated upon the decrease in podocyte number in several podocytopathies. We suggest the potential signaling pathways that may be triggered and regulatory processes introduced, revealing a significant role of PECs in the development and progression of podocytopathy.

## Author Contributions

Z-HL, X-YG, and X-YQ designed and wrote the manuscript. Z-HL and CY designed the figures. CY, Z-HL, Z-JL, H-YS, NA, and H-FL revised the manuscript. CY, NA, and H-FL obtained funding. All authors contributed to the article and approved the submitted version.

## Conflict of Interest

The authors declare that the research was conducted in the absence of any commercial or financial relationships that could be construed as a potential conflict of interest.

## Publisher’s Note

All claims expressed in this article are solely those of the authors and do not necessarily represent those of their affiliated organizations, or those of the publisher, the editors and the reviewers. Any product that may be evaluated in this article, or claim that may be made by its manufacturer, is not guaranteed or endorsed by the publisher.
